# Redefining neuromarketing as an integrated science of influence

**DOI:** 10.3389/fnhum.2014.01073

**Published:** 2015-02-12

**Authors:** Hans C. Breiter, Martin Block, Anne J. Blood, Bobby Calder, Laura Chamberlain, Nick Lee, Sherri Livengood, Frank J. Mulhern, Kalyan Raman, Don Schultz, Daniel B. Stern, Vijay Viswanathan, Fengqing (Zoe) Zhang

**Affiliations:** ^1^Warren Wright Adolescent Center, Department of Psychiatry and Behavioral Science, Northwestern University Feinberg School of MedicineChicago, IL, USA; ^2^Mood and Motor Control Laboratory or Laboratory of Neuroimaging and Genetics, Department of Psychiatry, Massachusetts General HospitalBoston, MA, USA; ^3^Applied Neuromarketing Consortium, Medill, Kellogg, and Feinberg Schools, Northwestern UniversityEvanston, IL, USA; ^4^Medill Integrated Marketing Communications, Northwestern UniversityEvanston, IL, USA; ^5^Department of Marketing, Kellogg School of Management, Northwestern UniversityEvanston, IL, USA; ^6^Aston Business SchoolBirmingham, UK; ^7^School of Business and Economics, Loughborough UniversityLeicestershire, UK; ^8^Department of Statistics, Northwestern UniversityEvanston, IL, USA; ^9^Department of Psychology, Drexel UniversityPhiladelphia, PA, USA

**Keywords:** neuromarketing, neuroeconomics, marketing communications, neuroimaging, scaling, influence, choice

## Abstract

Multiple transformative forces target marketing, many of which derive from new technologies that allow us to sample thinking in real time (i.e., brain imaging), or to look at large aggregations of decisions (i.e., big data). There has been an inclination to refer to the intersection of these technologies with the general topic of marketing as “neuromarketing”. There has not been a serious effort to frame neuromarketing, which is the goal of this paper. Neuromarketing can be compared to neuroeconomics, wherein neuroeconomics is generally focused on how individuals make “choices”, and represent distributions of choices. Neuromarketing, in contrast, focuses on how a distribution of choices can be shifted or “influenced”, which can occur at multiple “scales” of behavior (e.g., individual, group, or market/society). Given influence can affect choice through many cognitive modalities, and not just that of valuation of choice options, a science of influence also implies a need to develop a model of cognitive function integrating attention, memory, and reward/aversion function. The paper concludes with a brief description of three domains of neuromarketing application for studying influence, and their caveats.

## Introduction

Marketing has been dominated for over a century by models that assume a rational process of persuasion, which follows a sequence from awareness through purchase that consumers can consciously articulate. While this approach fits with traditional research methodologies, it hasn’t always explained or predicted purchase behavior. Recent developments suggest that a new perspective may be emerging. In particular, marketers have sought to integrate ideas about non-rational and rational processes (Kahneman, 2011), and ideas related to social neuroscience vs. individual decision-making (Lee et al., 2007; Senior and Lee, 2008), as well as using methods and technologies aligned with neuroscience (Ioannides et al., 2000; Braeutigam, 2005; Vecchiato et al., 2011; Plassmann et al., 2012). Some have been quick to label—not always in a complimentary manner—such developments as “neuromarketing” (e.g., Laybourne and Lewis, 2005,^1^).

To date, neuromarketing has lacked a solid theoretical framework. As such, the term “neuromarketing” itself runs the risk of confusing more fundamental scientific research with commercial applications (Lee et al., 2007; Javor et al., 2013). In this paper, we seek to extend existing work (e.g., Fugate, 2007; Hubert and Kenning, 2008; Senior and Lee, 2008; Wilson et al., 2008; Fisher et al., 2010), to clarify a framework for neuromarketing as an integrated science of influence. We start by contrasting neuroeconomics to neuromarketing. We then consider the concept of influence across individuals, groups, communities and markets, along with its dependency on an integrated model of mental function, along with some key—often unrecognized—caveats that must be considered by neuromarketing researchers.

## Influence vs. choice

**Figure 1:**
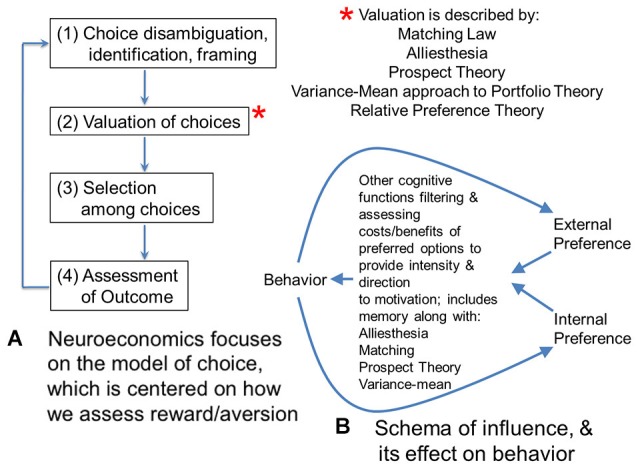
**(A)** Neuroeconomics focuses on the model of choice, which is centered on how we assess reward/aversion. This flow diagram shows four steps involved in making a choice. For the second step, there are several theories that have been proposed to model valuation of choices. Matching theory and alliesthesia (hedonic deficit theory) are two theories heavily used in neuroscience. Prospect and portfolio theory are used in economics. All four theories have been used in neuroeconomics. New to the set of valuation theories is relative preference theory (RPT) that is the only valuation theory meeting Feynman criteria for lawfulness, using an information variable, or actually scaling from group to individual behavior. Because of this scaling across group and individual behavior, and the fact it can be framed as a power law, RPT actually encodes the fundamental features of the other four theories, and can be used to ground them or even derive them. **(B)** In contrast to economics and neuroeconomics with their focus on choice, marketing is focused on “influence”, which looks at how distributions of choice behavior can be shifted or altered. This diagram sketches one potential model for the effect of influence on behavior. Influence can be considered the difference in gradients for preference inside a person (or organism) and outside a person. These gradients of preference might be schematized by RPT. They would be filtered and processed by valuation functions mentioned in panel **(A)**, which include alliesthesia or hedonic deficit theory regarding what is in deficit for an individual, along with matching, prospect theory, and the variance mean approach to portfolio theory. This processing would facilitate integration of the gradient inputs and determine what goal-objects or events become the focus of behavior, along with providing the intensity for it. Other cognitive functions such as memory are critical to this processing and evaluation of relative costs/benefits to prospective behavior; together they give behavior its direction and intensity. Behavior, in turn, feeds back onto these internal and external gradients of preference as experienced utility of expressed behavior.

It is helpful to compare neuromarketing to neuroeconomics, with which it may appear to overlap. Neuroeconomics tends to focus on individual and group choice, or judgment and decision-making in the context of consumables or markets (Figure 1A; Camerer, 2008). This focus on choice is distinct from the focus in neuromarketing on the issue of how individuals and groups might be shifted from one pattern of decisions to another pattern, or to change their *distribution of choices*.

Much neuromarketing research to this point has been focused on optimal methods to shift the distribution of choices (e.g., Ambler et al., 2004; McClure et al., 2004; Ohme et al., 2009; Santos et al., 2011). The use of “neuro” as a prefix has thus followed a similar rationale to that of neuroeconomics, whereby study of the neural processes provides a tool for describing behavioral change that was not available by the study of behavior alone (Ariely and Berns, 2010).

Such a view of neuromarketing ignores the broader perspective on what might be called “influence”, which is the primary issue involved with marketing, advertising, engineering design, teaching, or behavioral change in medicine. All of these categories of “influence” focus on how to get people to engage in a behavior preferred by a corporation, government, trade-group, culture or other entity. From an ethical perspective, discussions regarding consumer rights, for example privacy, are key when considering the influencing of behavior by interest groups, and neuromarketing research has been a subject of some interest in this regard (e.g., Murphy et al., 2008; Wilson et al., 2008). Nonetheless, influence doesn’t just shift the distribution of choices to one favored by the interest group in question, but balances between internal and external forces on behavior. Influence might be considered a balance between gradients of preference within an individual or group that influence events outside of them, and gradients of preference outside the individual or group that influence events inside of them.

Such gradients of preference could be schematized by patterns of approach and avoidance decisions (i.e., the distribution of choice) as described by relative preference theory (RPT; Breiter and Kim, 2008; Kim et al., 2010), an empirically-based account of reward/aversion resembling prospect theory (Kahneman and Tversky, 1979; Breiter et al., 2001) but grounded in information theory (Shannon and Weaver, 1949) to account for patterns in decisions that can be connected to reward/aversion circuitry and genetic polymorphisms (e.g., Perlis et al., 2008; Gasic et al., 2009). Using RPT, internal and external gradients of preference would involve variables quantifying (a) the pattern of approach decisions; and (b) the pattern of avoidance decisions. In the case of internal preferences, these would characterize the individual, whereas in the case of external preferences these might characterize a group of people external to the individual (or a preference gradient from just one other external person). RPT allows individual and group preferences to be readily characterized in a quantitative, lawful fashion that scales between individual and group. The integration of internal (e.g., individual) and external (e.g., group) gradients of preference would then be given direction in distinct decision/planning/problem solving situations by the processes briefly schematized in Figures 1A,B. Gradients of preference given direction by hedonic deficit theory (i.e., alliesthesia; Cabanac, 1971; Paulus, 2007) and other valuation processes (necessary for incorporating probabilities related to goal-objects, Kahneman and Tversky, 1979; relative valuations across goal-objects, Herrnstein, 1961; and variance in valuation, Markowitz, 1952) would constitute the combined intrinsic and extrinsic motivation described by Deci and Ryan (1985), leading to behavior, which in turn feeds back into gradients of preferences based on the experienced utility in individuals involved (see Kahneman et al., 1997). Such a schema is shown in Figure 1B as one of many possibilities for how internal and external gradients are balanced through their effects on behavior, and can shift distributions of choice.

**Figure 2:**
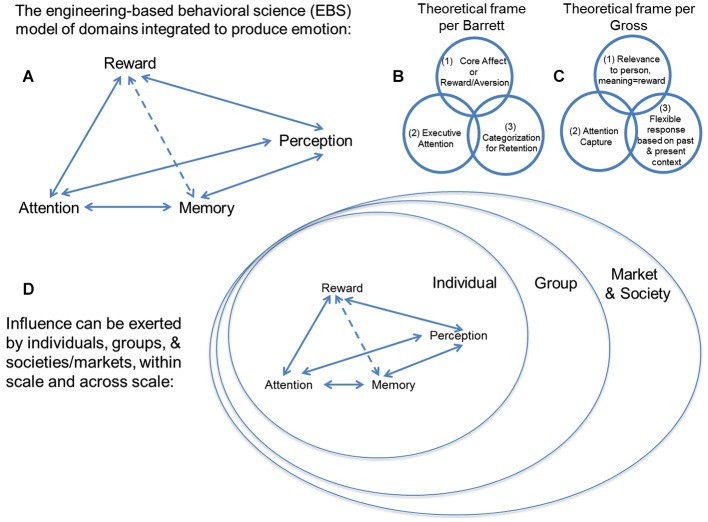
**(A)** This schema describes an engineering-based behavioral science (EBS) model of psychological domains that can be integrated in accordance with existing non- engineering-based models of emotion. Unlike other frameworks, EBS evaluates mathematical, law-like relationships between cognitive domains such as (i) reward/aversion processing, (ii) attention, (iii) memory, and (iv) perception, rather than associative relationships based purely on statistics. There are a number of modern theoretical constructs for emotion, including two examples shown from work by **(B)** Barrett and **(C)** Gross, and they tend to include the components we suggest integrating through EBS. **(D)** Individuals, groups, and/or societies/markets can exert “influence” to shift distributions of choice behavior in others. This expression of “influence” can be exerted within scale and across scale (e.g., by a group on an individual). Neuromarketing uses the valuation aspect of neuroeconomics (i.e., reward/aversion processing) and tries to integrate it with other behavioral science and neuroscience constructs, such as attention, memory and perception, which are all components of the EBS model. It tries to do this at the level of the individual, the group, and society, which are each different scales of measure.

In considering the balance between internal and external preference, cognitive processes thought to be separate from that of the valuation of options come into play, such as perception, attention, and memory (Ioannides et al., 2000; Ariely and Berns, 2010). At this time, no theoretical schema and little empirical data exist for how these theoretically independent cognitive processes interact, but cognitive processes for *perception* of outside stimuli exerting influence, *attention* to their features, and *memory* for comparison of such features to prior percepts are necessary operations for processing “influence”. Exerting “influence” to change another’s behavior, or being the subject of outside “influence” to change your own behavior thus need to be considered in a much broader construct of mental operations (see Figure 2A). One must also recognize that this ensemble of operations (i.e., perception, attention, memory, reward/aversion processing) have been extensively theorized to be core processes for emotion (Breiter and Gasic, 2004; Barrett, 2006; Gross, 2009, 2013; Kuppens et al., 2013; Lindquist et al., 2013). Specific examples of this are schematized in Figures 2B,C for the models of Barrett (2006) and Gross (2009, 2013).

The balance between internal and external forces on behavior (e.g., respectively, internal emotional experience (or internal preference gradient) vs. emotional expression by entities outside the individual (or external preference gradient)) must also be apparent at the neural level of measurement, given that “brain and mind are one”, a fundamental hypothesis of neuroscience (e.g., Breiter and Rosen, 1999; Breiter and Gasic, 2004; Breiter et al., 2006). This view of neuromarketing thus has as its focus an understanding of the balance between internal and external preferences (emotional experiences), on individuals and groups. Neural measures of one individual or interacting individuals (e.g., as with joint trust games; King-Casas et al., 2005; Tomlin et al., 2006) can be made in parallel with behavioral ones to confirm that observations made at the behavioral level affect those at other levels of spatiotemporal organization, or actually scale across levels of spatiotemporal organization (i.e., group behavior, individual behavior, distributed neural groups, neural group, etc.). Influence can thus be thought of as being present across multiple spatiotemporal levels of measurement, from group measures to measures of individual behavior to neural groups, etc. The issue of scaling might be considered as a “layering of influence” and warrants further discussion.

## Layers of influence and communities affected

Scaling is rarely discussed in experimental psychology and other behavioral disciplines, and was not formally introduced into behavioral science and neuroscience until the 1990s by Sutton and Breiter (1994). In its adaptation to biology and behavior, scaling refers to how measures made at one level of spatiotemporal organization, relate in a principled, lawful manner to measures at other spatiotemporal levels of organization (Sutton and Breiter, 1994; Perelson et al., 2006; Savage and West, 2006). This relationship does not represent a statistical one where a certain amount of the variance at one level of measure can predict the variance at another level, or how some information at one layer of organization can specify some extent of information at another layer (Adami, 2004; Szostak, 2004). Instead, it is causal (i.e., mechanistic) in that the same patterns of behavior measured at one layer are also measured at a neighboring level, and there is a necessary relationship (Sutton and Breiter, 1994; Breiter and Gasic, 2004; Breiter et al., 2006). Given that it has not been a major topic in behavior science or neuroscience, few biological measures have yet been shown to scale. One behavior that does show scaling is that of circadian rhythms, which show measures that scale from behavior to distributed groups of neurons to individual neural groups to cells and molecular biology. The other is approach/avoidance behavior described by RPT, scaling from group behavior to individual behavior, and potentially to other scales (Breiter and Kim, 2008; Kim et al., 2010). To date, few behavioral constructs outside of RPT have been tested to Feynman criteria for lawfulness, which includes scaling (Feynman et al., 1963).

Scaling has become an important metaphor/analogy in considering the statistical association of measures made at one spatiotemporal scale vs. another, as with the Research Domain Criteria project (RDoC) sponsored out of the National Institutes of Health (NIMH; Insel et al., 2010; Morris and Cuthbert, 2012; Cuthbert and Insel, 2013). The RDoC project and projects sponsored out of the National Institutes of Health Connectome Project (Van Essen et al., 2012; Barch et al., 2013) focus on measures at different spatiotemporal scales that can predict some degree of variance in each other. Both the RDoC and Connectome projects are directly modeled after the Phenotype Genotype Project in Addiction and Mood Disorders (PGP),^2^ which successfully discovered measures that scale across levels (i.e., RPT; Breiter and Kim, 2008; Kim et al., 2010).

While scaling is a *sine qua non* of classical science across levels of spatiotemporal organization, constructs that have become fundamental to more contemporary approaches to science have also become active considerations in neuroscience, in particular the issue of uncertainty (Kahneman and Tversky, 1979; Knill and Pouget, 2004; Gallistel and King, 2009; Kim et al., 2010; Vilares and Kording, 2011). Scaling and uncertainty are of interest with regard to neuromarketing, in that there is a common intuition that influence occurs between individuals, between individuals and a group, and between groups. As schematized in Figure 2D, influence is thought to occur in the interaction between individuals, who are embedded within groups, so that they affect their respective groups, and the larger framework (e.g., society, market) in which that group exists. This embedding of individuals/groups can be directly analogized to the embedding of networks (Sutton and Breiter, 1994).

This model of influence across scales of organization (e.g., individual, group, society/market) also relates to issues of uncertainty due to information loss in the communication between individuals/groups, or the uncertainty related to imprecision in the interpretation of communicated emotions (e.g., Figures 2B–D). Characterizing influence by scaling and uncertainty has some appeal, but begs the issue of what influence is in this context. When one considers influence in this model, it comes across as resembling a field of sorts, with a gradient of effects as two entities wielding influence come in greater proximity to each other (Figure 1B). To date, there has been no formal definition of influence, either through axiomatic derivation, or through iterative modeling (Banks and Tran, 2011) of behavior data to show a specific mathematical formulation of a pattern in a graph. Such work is clearly needed, and likely will depend on the cognitive processes identified to underlie this “field” of influence, such as those involved with emotion, discussed previously.

One might consider influence, and its potential scaling and effects of uncertainty, as a product of human psychology and the sub-processes underlying human information processing. Such considerations point to the importance of having a complete model of mental functioning, which is as yet lacking. Neuromarketing investigations can have a major input into the development of this integrated model, if they are conducted from a consistent and coherent theoretical base as discussed herein.

## Basing influence on an integrated model of mental function

At this time, we have no unified model of the mind, which shows how sub-processes such as attention, memory and reward/aversion processing are integrated and function concurrently for decision-making, planning, and problem solving. When one opens any cognitive science/biological psychology textbook, one finds chapters on information theory, perception, attention, decision-making, etc., but nothing integrating them. Even the use of the term information theory—although considered the basis of cognitive science—was never used in its mathematical framework in cognitive science until approximately 4–6 years ago (Breiter and Kim, 2008; Tononi, 2008; Gallistel and King, 2009; Kim et al., 2010). For the most part, marketers have relied on thinking about judgment and decision-making in terms of cognitive biases and mental functions involved with choice.

Recently, attention has been given to the building of such an integrated model of mental processing, starting with efforts to look at the input end of cognitive function, and to consider how quantitative descriptions of processes for reward/aversion, attention, and memory might work together. This work has led to research (Viswanathan et al., Under review) integrating parts of RPT (representing reward/aversion) with variables from signal detection theory (representing attention), and combining signal detection theory with Ebbinghaus memory functions to unpack sub-processes mediating working memory (Reilly et al., Under review). This early work argues that cognitive science constructs can be integrated, and points to the large amount of work needed to develop a comprehensive merger of domains in cognitive science, including domains such as decision-making, planning, and problem solving, along with output of the system in terms of motor behavior, language, and autonomic functions.

The ultimate integration of these cognitive functions can be analogized to a wall chart in biochemistry where all chemical pathways underlying biological metabolism are organized. We are a long way from having such an integrated platform for mental operations, particularly since such integrated systems as in biochemistry also convey mechanism and allow causal inference. In the short term, the viability of such an effort can start with developing complete constructs for attention, memory, and reward/aversion processing. Complete constructs for attention, for instance, would necessitate the mathematical description of the relationship between focused, selective, sustained, divided, and alternating attention. The potential for such complete constructs to be integrated across functions (i.e., perception, attention, memory, and reward/aversion processing) would then be a necessary second step in testing the viability of developing a general model of the mind.

Development of such a general model of mental function would allow us to theorize and empirically test what set of functions together respond to influence from another individual/organism, and exert influence on individuals/organisms outside of the person. With an integration of, at minimum, the functions thought to comprise emotion and memory thereof, cognitive psychology would likely be able to begin defining a quantitative model of influence. Such an effort will also depend on parallel assessments of the integrated cognitive model through approaches that (1) assess how well the integrated cognitive construct fits with neuroscience measures; (2) determine if important features of the construct can be derived axiomatically (an approach used extensively in traditional economics); and (3) test if the integrated cognitive construct facilitates the analysis of large data sets of human consumption and media use (referred to as “big data”), which should show features of human cognitive function.

Even so, efforts devoted towards developing neuromarketing as a science of influence, and towards a general model of mental function must remain cognizant of the risks inherent in such research, particularly given the persuasiveness of brain imaging (Roskies, 2008). Such risks are well covered in other foundational literature (e.g., Senior et al., 2011), but it is worth noting here that the subtractive and reverse inferential methodologies—predicated on observing specific brain region activity associated with specific tasks—are unable to conclusively confirm either the necessity of that specific region for that specific task, nor the lack of involvement of other regions, particularly in complex tasks (Friston et al., 1996; Poldrack, 2006, 2008). Confounds can also arise in neuroscientific studies of behavioral change (i.e., influence). It is a mistake to assume that one wants changes in both behavior and brain signal to interpret the effect of any influence. Such circumstances only lend themselves to interpretation when there is a parametric variation in both variables, which in turn can lead to a power problem. Rather, it is usually preferable for variables in *either* behavior or neuroimaging change, assuming the (often unrecognized) issue that baseline or comparative conditions remain unchanged also. Similarly, one must control for hormonal and demographic variables, which have been shown to influence key neuroimaging variables (Goldstein et al., 2005, 2010; Breiter et al., 2006). A final caveat is that we still do not understand the processes by which distributed groups of cells “process information” (e.g., Freeman, 2001). The functional domains of biochemistry, molecular biology, and genetics are quite distinct from those we hypothesize for behavior (e.g., attention, memory, reward/aversion processing, etc.), and how distributed neural groups produce functional domains and interact is far from understood. As such, all neural signals must be looked at as providing ancillary support for measures made at other spatiotemporal scales (e.g., behavior or genetics).

There also remain key issues in the use of “big data” approaches to neuromarketing. In particular, the high-dimensionality and huge size of data sets in this context can lead to inferential problems of their own—particularly spurious correlations, noise accumulation, and incidental homogeneity (e.g., Fan and Fan, 2008; Fan et al., 2013). The often uncontrolled and naturalistic collection of such data sets also has the potential to raise issues of public interest regarding the ethics of social research (e.g., Kramer et al., 2014). That said, as long as researchers approach their work in light of such caveats, big data provides opportunities for neuromarketing as a science of influence, in particular due to (i) its cohort sizes; (ii) its attention to demographic and socio-economic variables; and (iii) its broad array of variables that can be aligned to neuroscience variables.

## Summary

This manuscript provides a theoretical framework for neuromarketing based on the process of influence, and how it shifts distributions of choice across many scales of measurement, from individual to group/market and society. As opposed to issues of choice, issues of influence encompass a broader array of behavioral science domains, pointing to the importance of developing a rigorous quantitative model of mental function, which can provide testable hypotheses for how distributions of choice are shifted across scale and within scale (i.e., from individuals to groups/market to society and back again). However, a tremendous amount of work is needed to get to this point, and this work will need to meet the highest of academic standards if it is to change standards of practice and have real relevance for the marketing community and those involved with influence or behavior change, whether that be in education, medicine, business, marketing communications, design, or political policy.

## Conflict of interest statement

The authors declare that the research was conducted in the absence of any commercial or financial relationships that could be construed as a potential conflict of interest.

## Footnotes

^1^See Brain scam? (2004). *Nat. Neurosci*. 7, 683. doi:10.1038/nn0704-683; and Neuromarketing: beyond branding (2004). *The Lancet Neurol*. 3, 71. doi:10.1016/S1474-4422(03)00643-4.

^2^Lawler, A. “White house stirs interest in brain-imaging initiative”, Science, News, August 2, 2002; Abbott A. “Addicted”, Nature’s Senior European Correspondent, Nature, News Feature, October 31, 2002; and http://pgp.mgh.harvard.edu
